# The Role and Meaning of the Digital Transformation As a Disruptive Innovation on Small and Medium Manufacturing Enterprises

**DOI:** 10.3389/fpsyg.2021.592528

**Published:** 2021-06-09

**Authors:** Vasja Roblek, Maja Meško, Franci Pušavec, Borut Likar

**Affiliations:** ^1^Faculty of Organisation Studies in Novo Mesto, Novo Mesto, Slovenia; ^2^Faculty of Management, University of Primorska, Koper, Slovenia; ^3^Faculty of Organizational Sciences, University of Maribor, Maribor, Slovenia; ^4^Faculty of Mechanical Engineering, University of Ljubljana, Ljubljana, Slovenia

**Keywords:** digital transformation, disruptive innovation, Industry 4.0, Delphi study, SME, smart factory

## Abstract

The research reported in this paper explores the impact of digital transformation as a disruptive innovation on manufacturing SMEs. The research is based on a qualitative Delphi study encompassing 49 experts from eleven EU countries. The paper aims to demonstrate how disruptive innovations affect organizational changes and determine critical factors in organizations that impact the initiating and promoting R&D of disruptive innovation. We discovered that disruptive innovations impact product/process development methods, new production concepts, new materials for products, and new organization plans. Additionally, we identified organizational changes related to the development and use of disruptive innovations in the future. We also indicate how disruptive innovations influence social and technological changes in the organizational environment. The analysis also disclosed three main groups of disruptive innovations and their impact on future smart factory development, namely the following: technological changes, the emergence of innovative products, business models and solutions and organizational culture as one of the crucial key success factors. The analysis also examined the enablers of the successful development/introduction of disruptive innovations, wherein internal and external factors were determined. Additionally, we presented obstacles and the approaches necessary to mitigate them. We can conclude from the findings that in the timeframe of 5–10 years, only the SME that uses/develops disruptive innovations will survive in the market. However, the companies do not always have a clear idea of the meaning of disruptive innovations. Therefore, it is important to set clear goals regarding the achievement of disruptive innovations in companies. It is also necessary to creatively apply presented instruments enabling improvement of organizational changes and apply some additional concepts, which we have suggested.

## Introduction

The emergence of disruptive innovation theories dates to 1995, when Bower and Christensen (1995) published the article entitled Disruptive Technologies: Catching the Wave, which outlined the thesis that innovation drives corporate growth. Over the past 25 years, this thesis has become a guide for entrepreneurs and managers. Scholars usually ask why industry leaders do not remain leaders when technological or market changes occur. The answer can be found in the fundamental idea of disturbances theory as a tool that predicts behavior (Dillon, 2020). Its core value lies in the ability to evaluate and predict within the organization. The ability required by the organization is then one of choosing the right strategy and avoiding the wrong one (Shang et al., 2019). Such an instance is presented by the sale of the laptop IBM program to Lenovo, which is probably one of the most essential business decisions contributing to the continued growth and survival of IBM.

Disruptive innovations are defined as those based on which a product or service has been developed that incorporates a technology initially introduced in simple applications at a lower market price range (Christensen et al., 2018). These products or services are affordable in their original form. Disruptive innovations are not considered breakthrough innovations or ambitious upgrades of existing products or services that would dramatically change business practices and business models. Instead, they consist of straightforward and affordable products and services. Competitors recognize the market potential of such products and services, which are capable of transforming a particular industry. There is a knock-out effect of competition on the incumbent producers. They recognize factors of the primary producer (such as an internal organization) that prevent further product development and market penetration in compliance with predicted customer needs and expectations (Christensen et al., 2013; Dillon, 2020).

While work automation and computerisation were the critical paradigms of the Third Industrial Revolution (1960-2010), the Fourth Industrial Revolution (also named Industry 4.0) brought the digitalisation and informatisation of processes. Industry 4.0 can be understood as a broad socio-technical paradigm (Mariani and Borghi, 2019). It presents a policy concept for increasing economic growth, which has fostered the emergence of innovation-based entrepreneurship, and which is based on development and research, deregulation, increased risk capital financing and international protection of intellectual property (Christensen et al., 2018; Herrmann, 2019). The networking of the economy as a strategic tool for acquiring knowledge and information and connecting people with expertise in a modern knowledge society is crucial. The networking of businesses (e.g., incubators and technology parks) offers synergies in the joint management of information, knowledge and human resources. Knowledge and information become crucial for success in the Fourth Industrial Revolution (Kabir, 2019). The organization is required to do as much as possible, including optimizing resources, reducing costs per unit produced and enabling greater efficiency. Higher productivity with cost optimisation means a competitive organizational advantage. From the position of value and the value system, it is also vital to understand the current direction: striving for a balance between business and private life, a creative environment and the possibility of self-realization (Martin-Rojas et al., 2019). The new phase of evolution is connected with the development of the social superstructure and occurs only if suitable conditions are created in the broader social environment, namely the development level of information knowledge, individual consciousness, and attitude toward the environment (Nanterme, 2016; Bongomin et al., 2020).

During the Third Industrial Revolution, enterprises developed technologies that reduce cost and complexity. The development of technological processes has also enabled enterprises to produce more technologically advanced and higher quality products and services and develop new business models. However, in Industry 4.0, manufacturers are being challenged by the digital transformation, in which niche technologies, together with the Industry 4.0 concept, are understood as disruptive innovations. The most important developmental step within Industry 4.0 is establishing cyber-physical systems (CPS) which connect the physical environment and cyberspace (Ren et al., 2015; Lu and Xu, 2018). Within the systems, mechanisms are created that enable interaction at the human-to-human, human-to-machine and machine-to-machine level along the entire value chain (Kagermann et al., 2013). These processes affect changes in organizational culture and become an increasing challenge for companies and society, as the involvement of humans in the processes of direct communication and collaboration with the machine as an equal partner brings new challenges, such as the resistance of employees, the fear of replacing humans with machines and artificial intelligence-based technology, and the question of the adequacy of the skills necessary to manage organizational processes in the context of smart manufacturing (Hirsch-Kreinsen, 2016; Kiel et al., 2017; Seeber et al., 2020).

In manufacturing companies, the integration of CPSs into production creates cyber-physical production systems (CPPS) (Schiele and Torn, 2020). These systems become increasingly important in smart factories for creating connections along the entire supply chain (connection with suppliers – the company’s external environment) (Roblek et al., 2020). However, in the enterprise’s internal environment, changes in the production processes, wherein smart factory factors such as the industrial internet of things, CPPS and production systems consisting of one or more CPS come to the fore (Panetto et al., 2019). CPS is understood as a physical object with a built-in system in which the control process unit (computer power supply) is located, the industrial cloud, whose goal is to store, analyze and share data, with some form of network connectivity (Mabkhot et al., 2018). Thus, smart factories strive for self-organization based on establishing automatic machine configuration and process optimisation, enabled by the decentralization of production control. Innovative production process control software influences the transformation of shop floor management by introducing advanced technological processes based on lean management philosophy. For example, the Enterprise Resource Planning (ERP) at the planning level (top floor) uses objective performance data that captures all resources of the enterprise (shop floor) in real-time. The Manufacturing Execution System (MES) influences the improvement of production processes. It can connect production data and ERP data, including business planning that includes resources, customer requirements and expectations (Gruber, 2013; Oesterreider and Teuteberg, 2016).

In addition to CPPS, another characteristic of Industry 4.0 that influences the emergence of disruptive innovations within smart factories is that Industry 4.0 is based on and driven by technological development, represented by both self-oriented production manufacturing and service-oriented architects (Xu et al., 2018; Müller, 2019; Oztemel and Gursev, 2020). Technological development has influenced the emergence of smart products and services. It can be concluded that the result of Industry 4.0 is seen in the concept of smart factories, which is based on the intelligent production of smart, personalized products and within this production has a high degree of collaboration in production networks that also include external partners of the company value chain (Wang et al., 2017; Zhong et al., 2017; Frank et al., 2019).

The main objective of the research study is to identify disruptive innovations and understand their impact on future organizational agility. The paper also aims to present how disruptive innovations affect organizational changes and determine critical factors in organizations that impact the initiation and development of disruptive innovation. We focused on small and medium manufacturing enterprises (SMEs) in the European Union.

Based on these future expectations, the following research question was established:

Research Question: *What organizational changes should be expected from SMEs that enable the development and implementation of disruptive innovations and how do disruptive innovations pertaining to organizational changes influence future organizational agility?*

The following types of disruptive innovations were analyzed (and it has been estimated that they have an important impact on future smart factory development): (1) technological changes, (2) the emergence of innovative products, business models and solutions, and (3) organizational culture. These concepts enable manufacturing enterprises to reduce costs, improve flexibility and productivity, enhance quality and increase the speed of business processes (Brunelli et al., 2017; Junaid, 2020).

The research was conducted in European SMEs because micro, small, and medium-sized enterprises represent 99% of all European Union enterprises (Müller, 2019). The European Union promotes SMEs through various action programs, thus co-financing research programs in SMEs, which enable them a higher level of innovation and competitiveness (Hessels and Parker, 2013). Thus, SMEs have become the most propulsive companies in the EU and represent the European economy’s backbone (Dabić et al., 2016).

The paper consists of the following sections: introduction, followed by conceptual background (theoretical review). The third section includes methodology. The fourth section presents the research results. The paper concludes with a discussion of results and conclusion, including paper limitations, and proposes research in future development trends.

## Conceptual Background

The digital transformation in organizations is changing technology and business models. It brings challenges and opportunities for established companies and newcomers in the field of disruptive innovations. One of the most relevant results of the Fourth Industrial Revolution is the smart factory. The transformation of the classical factory into a smart factory begins with the digital transformation, measurements and informatisation of everything related to production systems. However, the development and implementation of Industry 4.0 niche technologies [advanced robots, additive manufacturing, augmented reality, simulation, horizontal and vertical system integration, the Industrial Internet of Things (IIOT), cloud computing cybersecurity, big data and big data analytics] for a manufacturing enterprise represents a disruption to the innovation that is transforming production (Brunelli et al., 2017). For example, Bruer et al. (2018) and Tortorella et al. (2018a) examined the connection between lean manufacturing and Industry 4.0. Ben-Daya et al. (2017) gave attention to the connections between the Internet of Things (IoT) and supply chain management. Liu et al. (2014), Li et al. (2017) and Oettmeier and Hofmann (2017) pointed out the influence of additive manufacturing on processes and performance in the supply chain. Ivanov et al. (2016) presented a dynamic model and algorithm for short-term supply chain in smart factories. The short-term smart factory supply chain is by their opinion based on “temporal machine structures, different processing speed at parallel machines and dynamic job arrivals.” New research regarding supply chain management research (Chang et al., 2020; Venkatesh et al., 2020) focuses on blockchain technology and its disintermediation effects. However, niche technologies as disruptive innovations also influence the organizational culture (Sultan and van de Bunt-Kokhuis, 2012; Tortorella et al., 2018b). Based on previous research into disruptive forces occurring in the industry, five crucial manufacturing disruptive methodologies that enable smart manufacturing can be highlighted. These five disruptive forces are (Li, 2016; McKinsey & Company, 2018):

(1) Connectivity-driven business models: The development and widespread availability of Internet technologies in the 21st century have made connectivity an essential factor in the emergence of new business models, among which the monetisation of data is a significant challenge. It is characteristic of the age of digitisation that software has become much more important than hardware. Interaction with customers is increasingly digital, in many cases managed without intermediaries, and takes place via digital industry platforms such are Amazon Web Services or, in the automotive industry, Mercedes Me Connect or Lexus Enform. Intel enables organizations implementing IoT solutions to connect almost any type of device to the cloud through their system architecture. It does not matter whether the device is connected to the native internet. IBM Watson technology platforms offer companies the opportunity to extend cognitive computing to IoT, and Microsoft Azure IoT platforms help companies to connect devices, prepare an analysis of previously unused data, and integrate business systems (Ionut Pirvan et al., 2019). Gawer and Cusumano (2013, 417) defined industry platforms as “products, services, or technologies that act as a foundation upon which external innovators, organized as an innovative business ecosystem, can develop their complementary products, technologies, or service.”
(2) Artificial Intelligence and autonomous systems: industrial companies are increasingly investing in robotics and machine learning. These investments enable them to develop technologies that enable the further development of the company’s core activities (for example, the development of an automatic vehicle for transporting materials and products within the company) (Roblek et al., 2020). Thus, learning data and developing intelligent algorithms becomes a competitive advantage for companies. The development of artificial intelligence and autonomous systems, both concerning production and incorporation into products, has already had and will continue to have an even more significant impact on the entire industry (Oztemel and Gursev, 2020).
(3) Internet of Things (IoT): the basis for evaluation, integration and optimal process control is process-related data. The data is obtained from measurements performed by different sensors (IoT). Intelligent sensors with an integrated microprocessor play an essential role in measuring and enabling their rapid digitalisation. Integrated intelligent sensors enable the execution of logical functions, two-way communication and adaptation to environmental changes, decision making, self-calibration and self-testing in start-up situations. The sensors are becoming smaller and more user-friendly. The IoT can be described technically as a combination of sensors such as RFID, other communication devices (i.e., embedded computers), CM applications, Enterprise Resource Planning (ERP) integration and business intelligence technology (Mabkhot et al., 2018). It is essential in manufacturing to expand the role of IIoT, CPPS and production systems consisting of one or more CPS. The CPS represents a physical object with an embedded system containing a control processing unit (computer power), the industrial cloud that can store, analyze and exchange data, and form a network connection. The emergence of CPPS in any production system enables economic, social and even ecological benefits (Thiede et al., 2016). McKinsey Global Institute predicts that the IoT potential is 10–20 percent energy savings and a 10–25 percent improvement in work efficiency (McKinsey & Company, 2018). However, according to casual theory, the question arises as to whether big data eliminates the need to search for causality? Here, it is necessary to first pay attention to the fact that organization data does not represent the phenomenon itself, but it is necessary to understand it as representational of this phenomenon. The purpose of providing continuous research within organizations, communities, and individuals is to reveal new insights by creating new data within new categories. It is necessary to be aware that big data overlaps or neglects irregularities unless we enable this with a search-analytical algorithm. The problem is that big data is much more focused on correlation than on causality and thus ignores average events or conditions (Song and Taamouti, 2019; Wamba et al., 2020).
(4) Electrification: the Fourth Industrial Revolution concerns the sustainability aspect of production and the environmental aspect, and the technical aspect of converting fossil energy to renewable energy and resource efficiency. However, environmental legislation and customer demand for sustainable products and services are forcing the industry to manufacture products that use electricity (e.g., electric cars) and other renewable energy sources (Moldavska and Welo, 2019).
(5) Cybersecurity: the increasing connectivity both within companies (man to machine and machine to machine) and between companies (company to company), companies and consumers (company to the customer) and other systems such as defense, transport, and banking reminds us of the importance of cybersecurity. As more and more closed systems open, there is a more significant threat to both work and property processes (such as industrial espionage). It is estimated that the cybersecurity market’s annual growth will be 5–10 percent by 2025 (McKinsey & Company, 2018). Companies have, therefore, begun to introduce the skills required for cybersecurity. Particular views of industry leaders suggest that they see cybersecurity as a battlefield for competitive advantage and diversity (McKinsey & Company, 2018).

Digitisation and informatisation enable the connecting of (smart) factories with other smart infrastructure elements – people, machines, and products. It is about connecting the entire value chain throughout the lifespan. People are involved as customers, constructors, technologists, managers and enhancers, repairers and analysts (Zhou et al., 2018). It can be concluded that connectivity enables organizations to adapt their systems to the needs of their customers in all aspects, specific requirements, quantities, deadlines and delivery points. The main challenges that organizations face in the digital transformation framework are standardization, security, and IT infrastructure. The real establishment of mentioned elements in the broader industrial environment will take several years, which is why some prefer to use the word evolution instead of the term ‘industrial revolution’ (Alvarez-Pereira, 2019).

In the context of research in the field of various manufacturing companies (breweries, automotive, food, textile, footwear industry, etc.), various authors (e.g., Yoo et al., 2012; Nosalska et al., 2019; Osterrieder et al., 2020) note that, in the context of Industry 4.0, digital transformation is coming which will lead to the emergence of smart factories. The digitalisation of production also affects customer requirements and business model change, the emergence of the digital (smart) supply chain (Garay-Rondero et al., 2020; Schniederjans et al., 2020), additive manufacturing technologies (D’Aveni, 2018) and increases the competitiveness of companies. The importance of disruptive innovations are noticeable in the context of full automation, robotisation and the development of manufacturing technologies that allow a higher degree of interconnectivity (IIoT), leading to increased communication between machines and local data processing. The research conducted in various German manufacturing industries shows that the machine and plant engineering companies are mainly facing changing workforce qualifications, while the electrical engineering and information and communication technology companies are mainly concerned with the importance of different critical partner networks, and automotive suppliers predominantly exploit IIoT-inherent benefits in terms of increasing cost efficiency (Arnold et al., 2016).

Hamzeh et al. (2018) researched the importance of technology and the Industrial Revolution concept for SMEs. The research was conducted among SME consulting managers who believed that technological development based on Industry 4.0 technology innovation would impact production costs, improve agility, and enhance service offerings. It should be noted that this is only a prospective study carried out among a very heterogeneous group of SME consulting managers. Chan et al. (2019) were attempting to determine how SMEs achieve the agility to respond to disruptive digital innovation. Their findings show “that for SME; mitigating organizational rigidity is enabled by the mechanism of achieving boundary openness while developing innovative capability is enabled by the mechanism of achieving organizational adaptability. At the same time, given the inherent challenges of resource constraints, SMEs also need to balance the tension of organizational ambidexterity”.

The transformation of traditional factories into smart factories will provide new insights into how disruptive innovations technology affects business process transformation, agility, value chain transformation, organizational culture, and human resource policy changes (Loonam et al., 2018). However, management in organizations must be aware that organizational and business issues remain the same in the age of smart organizations. The forces that cause disruptions are constant and affect both the internal and external organizational environment (e.g., supply chains which are transforming in the value chains) (Akkermans and Van Wassenhove, 2018). To ensure the successful operation of organizations and their long-term existence, leaders (often founders or significant shareholders) must provide adequate resources in the form of tangible and intangible assets. Therefore, they must be aware of the importance of acquiring knowledge that will enable the organization to cope with disruptive events and form a foundation on the basis of which management will be able to react to disruptive forces in a timely manner and provide a system for continuous management of disruptive events (Jaques, 2017). In doing so, the management must be aware of the importance of disruptive innovations theory and, on this basis, be able to predict what will happen without the hindrance of personal opinions (Wördenweber and Weissflog, 2006).

Organizations that want to be successful disruptive innovators must embed in their organizational culture the mindset that disruptiveness is not the creation of something new or breakthrough and that disruptive innovations are not events but a process in which resources are allocated within the organization, with a view to continuous technological evolution and meeting the changing needs of existing and potential new consumers (Rastogi et al., 2019). As part of its strategy, management must be aware of the importance of disruptive innovations policies within the Fourth Industrial Revolution. To this end, the organization’s strategy includes the importance of developing and adapting the system, organizational culture, organizational processes and other factors that enable the provision of fluidity even under reduced innovation conditions (Jaques, 2017; Hopp et al., 2018).

Szymańska (2016) and Mohelska and Sokolova (2018) explained that for ensuring success in the new work environment created by the Industry 4.0 era, it is crucial that organizational culture must be characterized by openness to various fields of activity. A new type of culture requires a new, open system of values, standards, thinking patterns, and actions perpetuated in the organization’s social environment, and contributing to its goals. The organizational culture in the Industry 4.0 era is primarily open to the environment, supports extensive cooperation therewith, provides freedom of relations, uses the potential of employees and external partners, and is open to new knowledge, changes, and sometimes to the resulting mistakes. Moreover, it focuses on implementing unique visions and strategies while ensuring discipline and successfully integrates participants in the described relationships around new activities (Al-Haddad and Kotnour, 2015).

## Methodology

### Delphi Methodology

Most Delphi researchers focus on the reliable and original research of ideas or advancing new information, which is useful in making important (strategic) decisions. Delphi studies are often used in deductive research but can be combined with data collected with qualitative methods that ensure a more pragmatic approach to instrumentalisation (Rowe and Wright, 1999). Consequently, this approach also allows for methodological triangulation (Yin, 2002), improves validity (Yin, 2013) and increases contextual understanding of the phenomena (Fiss, 2009).

The Delphi method is used particularly for predictions and forecasts concerning the future development of technology and the impact of new technologies on society and the economy. It is based on the statistical processing of collected opinions obtained from experts in a specific field. The Delphi method is a structured scientific method with clear rules and procedures. The experts are asked to answer some pre-selected questions, each on its own, and then the “average answer” is calculated. It is assumed that there are no “correct” answers, but the approach results in a free estimation of the probability that some events will occur. After collecting, processing and submitting answers to the same questions, definitive predictions are made (Higgins, 1994). The Delphi method’s key features are anonymity among survey participants, structured feedback that experts receive after giving opinions and allowing them to adjust their previous opinions until they reach an agreement (Hsu and Sandford, 2007). Usually, the Delphi method involves two to three rounds of exchange of opinions between experts and the researcher (Adler and Ziglio, 1996). Two are considered adequate (Boulkedid et al., 2011; Gary and Heiko, 2015) as the addition of further rounds adds a further administrative burden and places pressure upon participants that results in lower response rates (Gary and Heiko, 2015).

According to Loo (2002), the Delphi method can be used to forecast the future for strategic management and organizational development, among other potential applications for organizational management. Okoli and Pawlowski (2004) explained that the Delphi method was recognized as a widespread instrument in information systems research to identify and evaluate executive decision-making issues. Hallowell and Gambatese (2010) imply that Delphi technology is used in construction engineering and construction management when conventional methods fail because the latter may not be suitable for research involving disruptive factors and require sensitive data access. The Delphi technique is valued in such cases because it enables researchers to obtain highly reliable data from certified experts through strategically designed surveys. For this reason, we have chosen the Delphi method for our research. It helps us to establish procedures for obtaining and refining expert and professional opinions in the field.

### Delphi Study Design

The survey was conducted in two rounds. The Delphi study’s first round includes open-ended questions about expectations pertaining to the introduction of disruptive innovations in an organization, challenges experienced in introducing disruptive innovations, and steps for a successful introduction of disruptive innovations. The survey questionnaire was prepared in accordance with the questionnaire used in the MIT Sloan Management Review and Boston Consulting artificial intelligence survey (Boston Consulting Group, 2020). The questions were modified in accordance with the disruption innovation theme of our research. We tested the questionnaire on a sample of 12 persons that we had previously used in the survey. Following the comments of the participants, some minor mistakes have been addressed and complementary material was added to some questions in accordance with the topic of the research.

The questionnaire with four open questions was prepared in a survey tool named One Click Survey or 1KA (One Click Survey, 2021), and a link to the questionnaire was sent by email. The first question was: What disruptive innovation have you introduced into the organization and your strategy for further development? The second question was: What effect has disruptive innovation had on your organization so far? The third question was: What organizational changes do you think would result from disruptive innovations in the future (5–10 years)? The fourth question was: What are the key factors in the organization’s internal and external environment that enable further development and disruptive innovations?

Participants were given 10 days to provide their opinion and share expertise insights. Answers to open-ended questions were analyzed using qualitative content analysis. We informed the research participants of the results and allowed them to familiarize themselves therewith.

Based on the qualitative analysis of the answers obtained from round one of the Delphi study, seven expectations concerning the introduction of disruptive innovations in the organization were formulated. In the second round, participants were required to choose the appropriate answer in regard to introducing disruptive innovations in their organization. They were required to choose on the Likert scale the results they expected to achieve by introducing disruptive innovations. The third question includes ranking the predominant challenges that their company has experienced in introducing disruptive innovations. In the fourth question, they were required to specify the most important steps necessary to enable disruptive innovations. In the fifth question, they were asked to describe the importance and role of individual cultural values in developing and implementing disruptive innovations in their company. In the sixth question, they were required to list cultural values by their relevance to disruptive innovations in a changing environment.

We sent the questionnaire prepared using the 1KA tool in the second round to the participants who had answered all the open questions. All survey participants were given 14 days to provide answers. After 1 week, a reminder was sent. At the beginning of the third week, we thanked all participants who had answered the questionnaire. So, it can be concluded that all procedures necessary to undertake the standard Delphi method were followed during the study (Linstone and Turoff, 1975).

A comprehensive approach to the concept of the Delphi method was used. The information concerning the system of criteria and their relative importance creates the conditions for improving the quality of the design of a multi-criteria decision-making basis. The official expert prediction of the qualification weighting criteria was achieved through a methodologically defined, organized and systematized harmonization of individual assessments using descriptive statistical processing of these assessments and predictions (Hsu and Sandford, 2007).

### Delphi Panel

For this study, an expert was considered to have a broad understanding of smart manufacturing with specific expertise in at least one of four functional areas: human resource management, information systems management, research and innovation, and manufacturing. To be selected, an expert was required to hold either a middle or high-level managerial position in a smart manufacturing company. Moreover, each expert was required to be accessible and interested in the research results.

### Participants

The selection of suitable experts is of special importance. For this reason, the systematic approach was applied to select the appropriate participants for the study. In the first step, within various projects regarding innovations, workshops were conducted which were attended by participants and experts in the impact of disruptive innovations on the small and medium manufacturing enterprises. A list of those experts was formed. In order to meet methodological prerequisites for the Delphi study, the sample of appropriate experts was selected by applying various criteria, i.e., both genders were included in the study, from different work position levels (from board members to operation workers), years of work experience, country, and educational level. As a heterogeneous group of experts reflects the positively cognitive biases of the participants (Winkler and Moser, 2016), an emphasis was placed on an adequate heterogeneity of selected experts. Overall, a total number of 92 experts was identified and invited to participate in the study. All of them were contacted. By the end of the study, 49 experts from eleven countries (Slovenia (14), Italy (3), Spain (2), Hungary (5), Croatia (7), Czech Republic (5), Austria (3), Sweden (2), Germany (7), and Malta (1)) had completed both rounds of the Delphi study. Therefore, the participants’ final sample is purposive and consists of two board members, fifteen managing directors, seven technology directors, seven heads of business units or department, eight experts, three consultants, and seven operation workers. Their SMEs are, on average, more than 10 years old, with more than fifty employees, and generate an average of 3.3 million EUR in revenues per year. The SME primary industry is manufacturing, and the primary activity is R&D or product development, project management, strategy management, general management or information technology. They all have experience in using disruptive innovations as disruptive innovations in production, disruptive innovations algorithms and techniques, or disruptive innovations tools as an end-user.

### Assumptions and Biases of the Delphi Participants

The expert panel composition was based on identifying, evaluating, selecting, and recruiting relevant research participants. There is no general rule about the size of a Delphi study panel. Thus, the size depends on the purposes of the researcher, the desired heterogeneity, and the availability of the research expert (Loo, 2002). Researchers in past studies have used the Delphi method with 15–35 participants (McMillan et al., 2016) and studies with 40–60 participants (Kent and Saffer, 2014; Roßmann et al., 2018). The panel size in this study belongs to the second group and includes experts in digital transformation and smart manufacturing, which has become a complex topic involving different structures and actors, and the number of experts in this field is increasing. In practice, it has been shown that composite panels allow for more accurate estimates, as opposed to more diverse views, thereby reducing the specific polarization of preferences and responses (Yaniv, 2011).

The study involved a large number of stakeholders performing different functions within smart manufacturing. We ensured that the experts came from different countries. Potential experts were identified based on a database search and a network approach. The selection criterion focused on knowledge about smart manufacturing and the practice of a profession in this field. The experts were required to make appropriate statements about the importance of disruptive innovations and their future significance in the context of smart manufacturing. In the next step, we evaluated the experts regarding corporate functions and the importance of disruptive innovations in their smart factory environment.

## Research Results

The results of the study are presented in this chapter. Thus, the final rankings are shown, which were obtained based on the data analysis, and we added the explanations obtained through an analysis of the qualitative comments of the participants in the first phase of the study.

We decided to divide the research topic into two parts because a company’s digital transformation affects the emergence of change and the development of a new organizational culture. Thus, digitalisation in conjunction with the increasingly important informatisation represents an important field of research for the future, which includes not only technological changes in the field of final products (e.g., electric cars) and the robotisation of production and logistics processes (both have consequences for the supply and value chain and future employee structures of companies, etc.), but also raises the question of the emergence of a new organizational culture and leadership with increasing cooperation between humans and machines (Caruso, 2018). The first part addresses the impact of disruptive innovations on the organizations, while the second part presents the impact of the disruptive innovations on the organizational culture.

### The Impact of Disruptive Innovations on the Organizations

**FIGURE 1:**
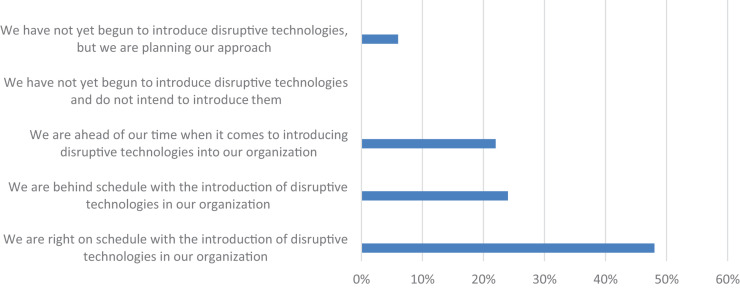
The level of introduction of disruptive technologies in the organizations (*n* = 49; source: authors).

In the second round of the Delphi study, the participants were first asked about adopting disruptive innovations in their organizations. Figure 1 shows that 48% of the participants think that their organization is on the right track with disruptive technologies, while 24% of the participants think that their organization is behind schedule with adoption and 22% of the participants think that their organization is ahead of schedule in adopting disruptive technologies and 6% of the participants think that their organization has not yet begun to adopt disruptive technologies but plans to do so. None of the participants thinks that their organization has not yet begun to adopt disruptive technologies and does not plan to adopt them.

The second question analyzed the % of participants expecting an increase in organizational performance by introducing disruptive innovations. Table 1 shows the listed outcome expectations in accordance with their importance for the participants.

**TABLE 1:** Results of participants’ SME achievement expectations by introducing disruptive innovation.

Expectations	1–10%	10–20%	20–50%	50–100%	100–200%	200–500%	500%>	Valid	Average	St. dev.
Increase revenue	9	14	12	8	5	1	0	49	2,80	1,35
	18%	29%	24%	16%	10%	2%	0%	100		
Increase market share by	17	12	8	5	6	1	0	49	2,60	1,53
	35%	24%	16%	10%	12%	2%	0%	100		
Reduce operating costs by	9	18	11	5	4	2	0	49	2,70	1,34
	18%	37%	22	10	8	4	0	100		
Increase business speed and agility by	8	13	9	10	6	3	0	49	3,10	1,49
	16%	27%	18%	20%	12%	6%	0%	100		
Improve customer satisfaction by	5	8	12	15	6	3	0	49	3,40	1,36
	10%	16%	24%	31%	12%	6%	0%	100		
Reduce the development time for new products/services by	10	16	7	5	3	8	0	0	3,00	1,73
	20%	33%	14%	10%	6%	16%	0%	100		
Improve amount of better talent hired and retained by	6	13	17	7	0	6	0	0	3,00	1,44
	12%	27%	35%	14%	0%	12%	0%	100		

*(n = 49; source: authors).*

According to the results in Table 1, the study participants indicated that they expect that 29% of the participants think that the introduction of disruptive innovation will increase sales by 10–20%. 35% of the participants think that there will be an increase in market share by 1–10%, and 37% of the participants think that operating costs will decrease by 10–20%. 27% of participants think business speed and agility will increase by 10–20%, 31% of participants think customer satisfaction will increase by 50–100%, 33% of participants think the new product/service development time will decrease by 10–20%, and 35% of participants think the number of more talented personnel hired and retained will increase by 20–50%.

**FIGURE 2:**
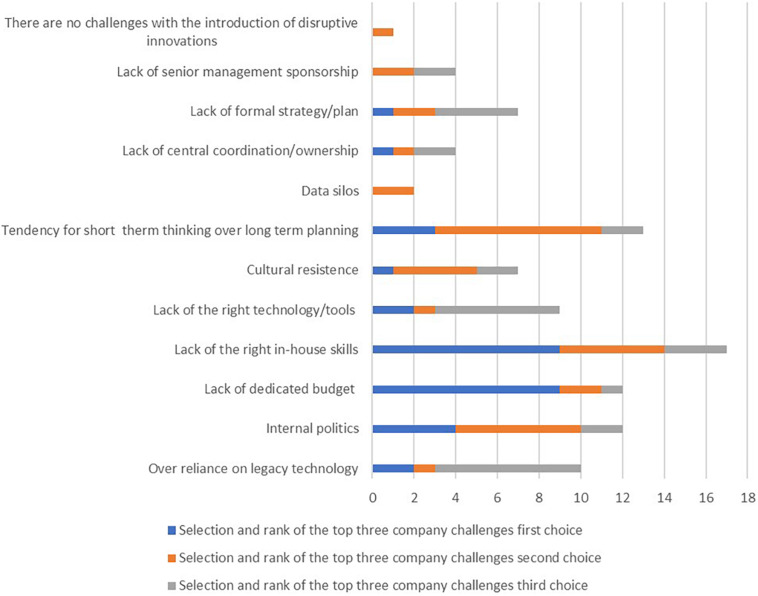
The average rank of the most important organizational challenges (*n* = 49; source: authors).

In the third question, participants were asked to identify and name the three most important challenges for their company in introducing disruptive innovations. Figure 2 shows that the most important challenges for companies in adopting disruptive innovations are the following: lack of the right in-house capabilities (11 votes), tendency to think short-term vs. plan long-term (7 votes), internal politics (5 votes), lack of a dedicated budget (5 votes), over-reliance on legacy technology (4 votes), lack of the right technology/tools (4 votes), cultural resistance (3 votes), lack of formal strategy/plan (3 votes), data silos (2 votes), lack of central coordination/ownership (2 votes), lack of senior management support (2 votes), and one participant indicated no challenges.

In the first part of the Delphi study, participants mentioned in their qualitative comments that a lack of the right technology/tools occurs in their organizations. However, participants do not pay much attention to this problem (or do not perceive it) because they lack the right internal skills and budget. They also mentioned that they have a higher-than-average tendency to think short term while planning long term. In the first part of the study, participants also pointed out the lack of a positive attitude among senior management regarding supporting technology implementation and helping employees overcome implementation or development challenges. Participants also believe there is a lack of central coordination in their organizations regarding ownership. In the qualitative comments, participants also pointed to issues related to over-reliance on outdated technology that, if not addressed, could lead to the creation of a dysfunctional organization. A culture of resistance may be associated with the challenge of a dysfunctional organization.

Concerning the challenge referred to as cultural resistance, the first part of the study examined which organizational culture values correspond to the adoption of disruptive innovations. It was found that the interplay between external and internal environments, technology orientation, and appropriate communication is of great importance. In the context of the development and use of Big Data, organizations are faced with the emergence of large volumes of unstructured data. Therefore, organizations must implement tools based on algorithms (e.g., Hidden Markov Model) to extract terms from data silos. Failure to address this challenge can lead to a dysfunctional organization. Within the internal policies, participants pointed out a lack of methods and procedures.

Participants in the Delphi study’s first part pointed out that disruptive innovations in business processes initially involve some resistance due to lack of internal knowledge, but this can be mitigated with the right methods. Some organizations had problems with employee resistance, especially with all methods, and needed more time for organizational change due to information support.

Participants highlighted the importance of digital transformation, enabling the introduction of new smart factory modules, technological improvements, robotisation (e.g., a laser camera system for seam tracking in mig/mag welding) and virtual (CPS) development. The consequences are apparent in the elimination of operators in the work process as such processes become modified by the deployment of robots. They also mentioned the importance of business methods such as Kaizen-5s and the implementation of 6 Sigma. According to them, disruptive innovations also change product/process development methods, bring new production concepts, new materials for products and new organizational plans (flat organizations, organizational flow changes, and more internal communication). For example, they enable greater effectiveness, real-time information for better decision making, fewer bottlenecks, seam tracking systems to enable better penetration and less dispersion of weld quality, and changes in supply chain management (e.g., the supplier can monitor inventory through online access).

The participants also emphasized that the strategic decision to engage in disruptive innovation is critical to success. Innovation does not arise from inspiration but from a clear, ambitious goal, business excellence, hiring the best talent inside or outside the organization, dedicated funding, and a strict timeline. Positive disruptive innovations include making the organization more agile and flexible. Other disruptive innovations, such as electric cars, bring some risks in the future and opportunities for a greater level of sustainability. Generally, if the disruptive program or product generates a significant cash flow, the organization must adapt to that opportunity.

Regarding the position of what organizational changes will occur in 5–10 years due to the development of disruptive innovations, the participants came to the encouraging conclusion that in 5–10 years, only the SMEs that develop disruptive innovations will survive in the market.

The participants’ comments included full digitisation, more virtual development, a different way of working, new offerings, new knowledge, new production concepts and market opportunities, shorter time-to-market, and collaboration between different market players. They also think that companies will have fewer staff, and supervisors with a higher educational level. Smart factories will need highly educated people and continuous updating of knowledge to manage their systems. Some participants also stated that the paradigm is changing dramatically right now due to the coronavirus, and it is difficult to predict what will happen in the future.

Participants expect that artificial intelligence will have an increased presence in business, especially in regard to big data. In the participants’ opinion, fewer people will require administrative or middle management, especially in middle-sized organizations. They asserted that the decision-making process must be quicker; development times for new products will be shorter; and the niches will become more critical because people will expect personalized products or services. The robotic lines will require different methods of guidance and monitoring. Reorganization of information support will be required, as will the increased awareness of line managers. It can be concluded that the business landscape will change drastically in the coming years as companies that are unwilling to adapt lose their market shares to new companies with new visions and monetisation approaches.

Participants ranked the most important organizational factors capable of enabling the further development of disruptive innovations in the internal and external environment as follows: the cosmopolitanism of the team, which can bring courage, openness and open-mindedness, which drives innovation, communication with people and their consultation, competition in the market, competitiveness, the desire for progress, new working methods, and the gathering of ideas. Helping top management to adjust and urge the adoption of high-level, open-source development toolkits allows a high level of abstraction and rapid development.

Among the internal factors that have proven to be the best and most effective in all aspects are openness to change, willingness to adopt new or innovative business models, organizational culture, budgeting, and external subjects’ willingness to participate. Among the external ones: competition (and cooperation, where complementary technologies are available or have the x-industrial application potential) and environmental friendliness (no safe/clean environment, no existential duration).

The organization’s expectations regarding the results achieved with disruptive innovations are based on the participants’ knowledge of the expected results of disruptive innovations in their organizations.

**FIGURE 3:**
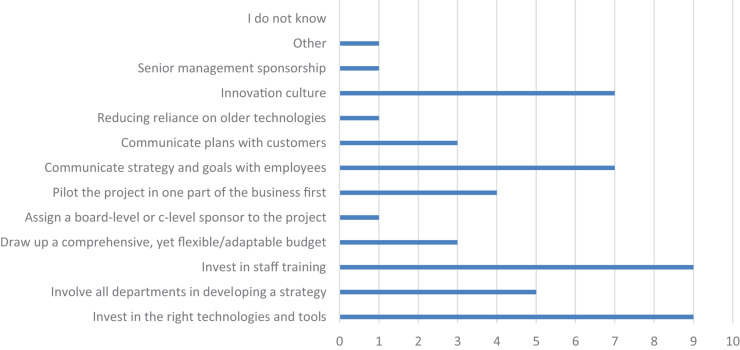
The most important steps to enable the successful introduction of disruptive innovations (*n* = 49; source: authors).

In the fourth question, participants were asked to mark the most important steps from the list that would enable the successful introduction of disruptive innovations. Figure 3 shows that the participants decided that the most important steps for a successful introduction of disruptive innovations are: investment in the appropriate technologies and tools, communicating strategy, investment in staff training, employee goals and innovation culture. Among the least important steps, participants ranked reducing reliance on older technologies and assigning a board-level or c-level sponsor to the project and senior management sponsorship.

The next subsection presents the answers regarding organizational culture changes in organizations due to disruptive innovations. We want to stress that the next subchapter is based on the same questionnaire (questions 5–6), which addresses the impact of disruptive innovations on the organizational culture.

### The Impact of Disruptive Innovations on the Organizational Culture

Development of the innovation culture is based on methodological knowledge of disruptive innovations. In question 5, the participants were asked to describe the importance and role of the individual cultural values in developing and implementing disruptive innovations in their organizations. The comments received in response to this question are added to the answers received in response to question 6.

**FIGURE 4:**
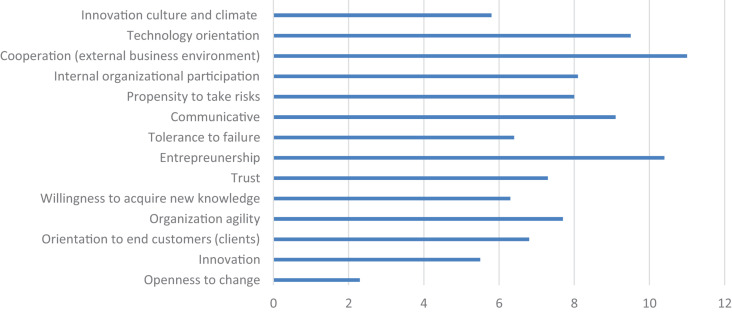
Cultural values by relevance, ranked from 1 (most relevant) to 12 (least relevant) in terms of their contribution to disruptive innovations in a changing environment (*n* = 49; source: authors).

In question 6, the participants were asked to rank the listed cultural values by their relevance in terms of their contribution to disruptive innovations in a changing environment and to provide a qualitative comment. The results are presented in Figure 4.

The cultural values listed according to their relevance are:

(1) *Openness to change*: The processes of change in SMEs are seemingly independent of each other, but the facts clearly show that they are closely interrelated. Specific social rules (e.g., legal, economic, and ethical) apply to each phase of change. It is important to be aware that change always has a deterrent effect on employees and that employees often feel threatened by innovations, which is why it is necessary to convince them of the benefits of change.
  For these reasons, the focus of leadership and management shifts spontaneously from functions and processes closer to direct relations with employees. Managers should be careful, when implementing organizational changes, to establish an appropriate work environment and rules and regulations, because only the efficient use of intellectual resources allows continuous improvement. It is appropriate to have such processes in a firm internal staff in a company that manages these processes.
  This distrust of employees toward the introduction of innovative solutions in the company requires that organizational change behavior should be encouraged at all levels of leadership, management, and implementation. Organizations need creative employees who can become involved in strategic thinking processes and can pass on new values, creativity and innovation to other colleagues.
(2) *Innovation approach, innovation culture and climate*: From the content analysis of the qualitative attitudes of the interviewees, it can be concluded that digitalisation and informatisation stand for the transformation of organizational processes through the use of innovative, disruptive technologies and solutions that will change the supply chain, technology, technological processes, the value chain and the future employee structures of companies. As it can also be seen from the next respondent’s answers, employees expect the emergence of a new corporate culture, increasing awareness of the importance of innovation and introducing new technologies and the interaction between management and employees for mutual cooperative cooperation in developing an innovative environment (including reach goals and creating a list of incentives for employees). Approximately 1–3% of company staff dedicates SME time for innovation, so it is important to stimulate and reward such staff (not only financially but also through other means of motivation – knowing individuals’ cultural values might help managers to obtain the optimum performance from an innovative team). It is important to note that the benefits of innovation are inevitable in the background of innovation culture. According to the respondents’ experiences, people were more inclined to embrace innovation if they saw a benefit to the individual. It is also important to emphasize that it is easier to manage an innovative company when managers and other employees originate from the same technical background because it is then easier to understand the situation in the market and transfer the appropriate knowledge for reaching the set goals. As part of developing cultural values for developing an innovative company, it is necessary to ensure that the natural curiosity of employees is maintained. It is also necessary to consider that better relationships promote the development of the company’s culture and climate toward unification, better understanding, and the achievement of its set goals.
(3) *Willingness to acquire new knowledge*: Companies must realize that in a modern organization in the Fourth Industrial Revolution, learning must take an active role in operations. Employees who want to educate themselves further to make the organization more sustainable must be encouraged to do so because further education is not connected with costs. The management should know that considerable benefit can be derived from having qualified employees.
(4) *Tolerance to failures*: the respondents point out the need to consider that mistakes occur in developing and implementing disruptive innovations. According to the respondents, intolerance to mistakes is the biggest obstacle to disruptive innovation. The reaction to mistakes also depends on the employee’s position, so the higher the decision-making level of a person, the more lenient the reaction to mistakes. However, learning experiences are never drawn from a mistake.
(5) *Orientation to end customers (clients)*: the respondents believe that customer orientation depends on the nature of the company’s products or services. However, awareness of empathy and listening to the customers helps in achieving/satisfying customer needs and thus improving the business.
(6) *Trust*: according to the respondents, “trust and security” are related, but they are also influenced by the “appreciation and treatment of the employee” by his superiors. Unfairness is just as detrimental to the issue of trust as it is undesirable.
(7) *Organization agility*: respondents indicate that their companies are dedicated to technological solutions and the openness of ownership/management structures to introducing new technologies.
(8) *The propensity to take risks*: risk-taking is evident in large new technology projects in organizations. Companies in which the culture discourages risk-taking become moribund. Innovation is 99% failure and 1% success.
(9) *Internal organizational participation*: internal organizational collaboration is carried out in accordance with employee rules and qualifications. If innovation is perceived as a process, and different departments participate in the development, then the innovation process is more effective and productive.
(10) *Communicative:* this pertains less to cultural values than it does to the nature of a person’s character – extroverted vs. introverted. However, certain environments can influence good communication and bad communication, so, in part, the community’s cultural values influence the form and scope of the communication action.
(11) *Technology orientation*: according to the respondents, it is an asset for a company if the owners/managers originate from a technical background: a vision/strategy that is built into the culture needs to be passed on to the other employees.
(12) *Entrepreneurship*: according to the respondents, no individual would become an entrepreneur if their attitude was not one that is oriented toward exploring opportunities. The difference in how to do so is grounded in moral-ethical standards, which are part of one’s cultural values (also derived from childhood). Certain respondents pointed out that entrepreneurship is tied to making money from innovation. Thus, it might be a good step if management can explain how an innovative entrepreneurial spirit in the company can increase profitability. Among other answers, it is worth noting then that many employees started their careers in start-up companies.
(13) *Cooperation*: two different relationships emerged between companies: cooperation vs. competition. It is typical for small high-tech companies to cooperate (otherwise, they have little chance of surviving in the larger market). From this point of view, the younger generation’s cultural values are somewhat different from those of the older generation or those of the larger companies in which there is a competitive relationship between companies. In a cooperative relationship with external companies, communication occurs at the level of the most qualified professionals.

The cooperative relationship is gaining importance because the innovation life cycle is becoming shorter, and companies cannot afford to develop everything themselves. Therefore, the involvement of external parties plays an important role (e.g., outsourced development of partial technologies, test procedures, supply chains, etc.).

Following the analysis of organizational culture factors and innovative SMEs, it is possible to form the key meanings of the individual roles of organizational culture. Thus, it is important for SMEs, which want to be leaders in innovative development that the leaders and managers of the company enable the knowledge and information to be shared between all key personnel as quickly as possible. Within the framework of enabling an innovation approach and the innovation culture and climate, it is necessary to ensure that the emergence of new technologies does not have a negative impact on employees (the issue of dismissal of employees). Thus, the key social capital must be represented by employees, who will be given support in the form of guidance and motivation supplied by the company’s management to dedicate themselves to development without possible existential threats. It is important that employees trust their managers and leaders. As part of knowledge management, which we understand as a long-term and complex process of knowledge creation, transfer, and use within an individual organization, companies must provide the function of knowledge transfer and use as we have already established and enable employees to have constant access to the acquisition of new knowledge. The company must therefore encourage and motivate employees to attend various forms of education. It is also important for an innovative organization to accept certain risks as one of the factors. Therefore, a certain level of attention must be paid to risk management and tolerance to the failures in R&D. The company’s technological infrastructure must enable the customer to fulfill almost every wish regarding the company’s products efficiently and with high quality. However, the technology infrastructure alone is not enough to fulfill the wishes of customers in the best possible way. Of course, the essential factor of the company philosophy must become an absolute focus on the customer and on the best educated and most highly motivated employees. Within the framework of organizational agility, both business owners and management must focus on permanent investment in new technologies. It is beneficial for the company if the owners and management support the technological orientation of the company, and define this in the vision/strategy of the company. An innovative entrepreneurial spirit is also encouraged in innovative organizations. In doing so, the company must provide employees who join the internal enterprise with payment outside the usual salary system in the organization. The employee must thus agree to a reduction in salary in the event of business failure, which is understood as entrepreneurial risk. In the event of success and generated profit, the individual is, of course, rewarded. An internal entrepreneur is, of course, different from a classic entrepreneur. The basic characteristic of an internal entrepreneur is that they are directed by the management of the company, while a classic entrepreneur is completely independent. The internal entrepreneur is also less risk-averse, but at the same time knows that in the event of failure, they will remain relatively safe within the company. Finally, we must mention the importance of developing a cooperative culture, which is important for creating a positive climate between individual organizations involved in the development or manufacture of a particular product.

## Discussion and Conclusion

A Delphi method was applied as a tool in order to identify points of agreement about disruptive innovations within a group of experts. The study’s goal was to determine the answer to the research question: *What organizational changes should be expected from SMEs that enable the development and implementation of disruptive innovations and how do disruptive innovations pertaining to organizational changes influence future organizational agility?*

This section will briefly summarize the key results and add the discussion, which illustrates the results and enables a wider picture and a comprehensive answer to the research question.

At the beginning of the research, the participants were asked how they define disruptive innovations. We discovered that participants have very similar definitions of what disruptive innovation means. The definition could be summarized as innovations based on developing specific and affordable products or services. They are not considered to be breakthrough innovations or ambitious upgrades of existing products or services. Into their organizations, they introduced, for example, the following disruptive innovations: several modules for the smart factory, different approaches to regular workdays, product innovations (e.g., products that reduce emissions in diesel gate engines), technological improvements (e.g., the technology that changes the production of components for electric motors), innovations of supply models and working processes.

In our opinion, a significant part of the identified and presented examples of disruptive definitions are only partially compliant with the basic definition of disruptive innovation. In the work of Christensen (1997), disruptive innovation is defined as something that creates a new value by disrupting existing value network(s), resulting in displaced dominating market-leading organizations or dominating products and services. Such innovations are more often than not produced by newcomers or even complete outsiders rather than existing market-leading entities. Moreover, “Disruption” often describes a process whereby a smaller company with fewer resources can successfully challenge established incumbent businesses (Christensen et al., 2013). The Disruptive Innovation is not each innovation, but those that significantly affect the way a market or industry functions.

Before continuing with a discussion, we shall provide a synopsis of the second part of the research findings. The participants pointed out that disruptive innovations in business processes initially bring some resistance due to a lack of internal knowledge but can be mitigated with the right methods. Some organizations had problems with employee resistance to all methods and need more time for organizational changes based on information support. Disruptive innovations impact product/process development method changes, new production concepts, new materials for products and new organization schemes (flat organizations, organizational flow changes, and more internal communications). So, they enable higher effectiveness, real-time information for better decision making, fewer bottlenecks, seam tracking systems enable better penetration and smaller spread of weld quality, and changes to the supply chain management (e.g., the supplier was allowed to observe inventories through online access).

Analyzing the “disruptive” innovation examples in this section we can realize that innovation examples are mainly not true disruptive innovations, but often improvements as a result of horizontal enabling technologies such as tracking systems and chain management tools, and ICT/digitalisation implementation. In other cases, the innovation was an implementation of widely accepted management models such as flat organization and improved internal communication. If we merge findings from this and previous paragraphs, it is clear that in many cases we detected a misunderstanding of the term ‘disruptive innovation.’ According to innovation typology (Nedelko and Potočan, 2013; OECD, 2021), respondents often presented process and organizational innovations which were new for the company but did not have the disruptive innovation character. This is compliant with the finding by Prof. Christensen that: “In our experience, too many people who speak of “disruption” have not read a serious book or article on the subject. Too frequently, they use the term loosely to invoke the concept of innovation in support of whatever it is they wish to do. Many researchers, writers, and consultants use “disruptive innovation” to describe any situation in which an industry is shaken up and previously successful incumbents stumble. But that’s much too broad a usage.” (Christensen et al., 2013).

Why are we stressing this issue? It is not the basic problem that the respondents do not know exactly what disruptive innovations are. It is more worrying that they might be satisfied with their innovation activities, believing that they properly manage disruptive innovations.

The last part of the summarized results presents the most important organizational factors capable of enabling the further development of disruptive innovations in the internal and external environment. These are as follows: the cosmopolitanism of the team, which can bring courage, openness and open-mindedness, which drives innovation, communication with people and their consultation, competition in the market, competitiveness, the desire for progress, new working methods, and the gathering of ideas. Helping top management to adjust and urge the adoption of high-level, open-source development toolkits allows a high level of abstraction and rapid development. Among the internal factors that have proven to be the most effective in all aspects are openness to change, the willingness to adopt new or innovative business models, organizational culture, budgeting, and external subjects’ willingness to participate. Among the external ones are competition (and cooperation, where complementary technologies are available or have the x-industrial application potential) and environmental friendliness (no safe/clean environment and no existential duration). Regarding the position of which organizational changes will occur in 5–10 years due to the development of disruptive innovations (third research question), the participants drew a satisfying conclusion that in 5–10 years there will be companies that develop disruptive innovations, while the rest will probably not survive in the market. The views regarding organizational changes that will occur in the future include full digitisation, more virtual development, a different way of working, new offerings, new knowledge, new production concepts and market opportunities, shorter time-to-market, and cooperation between different market participants. They also indicate that organizations will have fewer working staff, and supervisors with a higher educational level. Smart factories will require, for the purposes of managing their systems, more people with a higher level of education and continuous updating of knowledge. Some participants also state that the paradigm is currently changing dramatically due to the coronavirus and it is hard to predict what will happen. Participants expect that artificial intelligence will have an increased presence in business, especially in regard to big data, so that fewer people will be needed in administrative workplaces or middle management places, especially in larger companies. Decisions must made more quickly; the time to develop new products will be shorter; the niches will become more critical because people will expect personalized products or services. The robotic lines will require different methods of guidance and monitoring. Reorganization of information support will be essential, as will the increased awareness of line managers.

Based on these interesting research findings, we can make some conclusions. The first obvious finding deals with the business landscape, which is changing drastically and will continue to do some in the coming years. Companies that are not able or willing to adapt are losing their market shares to new companies with “disruptive” visions and monetisation approaches. We also estimate that companies are aware of present and future organizational challenges and mechanisms which are essential for a successful near future (5–10 years) organization, as presented in previous paragraphs. Our research results also reflect the idea of the Top 10 Skills of 2025, introduced by World Economic Forum (WEF, 2021). In addition to the presented key success factors, we would like to explicitly stress the Open Innovation and Triple/Quadruple Helix concept, which are already “a must.” Cooperation with academia is also an important tool for achieving disruptive and breakthrough innovations. Last, but not least, there are also methods available that enable the creation of disruptive innovations (Likar and Trcek, 2020). However, companies are, in our opinion, aware of the necessary organization culture instruments, representing prerequisites for disruptive innovations. But it is not enough to be aware of appropriate key success factors only. It is obvious that these must be applied in a creative and efficient way. Thus, the presented instruments can enable improvement of organizational changes.

Nevertheless, it seems that one aspect is missing – a clear understanding of the term “disruptive innovation.” Companies should understand what disruptive innovations are and set clear goals, i.e., more ambitious disruptive innovation development goals. Only in this way will they be able to focus their potential appropriately and perform all the necessary activities to achieve disruptive innovations and improve business results.

## Practical Implications

Based on the results, we prepared a set of practical implications for companies.

Firstly, a clear understanding of the term “disruptive innovation” is often missing. Companies should understand what disruptive innovations are - those that significantly affect the way a market or industry functions. Therefore, they should reconsider and set clear goals, i.e., more ambitious disruptive innovation development goals. Only in this way will they be able to focus their potential appropriately and perform all the necessary activities to achieve disruptive innovations and improve business results. A prerequisite is a clear vision of top management, which should be supported by concrete, clear and focused systemic changes and activities as follows.

It is important to develop employee competencies so that they feel confident to be ready for new challenges. One of the crucial competencies is the desire for progress, readiness to learn, prompt adoption of new working methods, and creativity/innovation orientations. In addition, the development of cosmopolitanism of the team is important as this can bring courage and open-mindedness, which drives innovation and competitiveness.

How to achieve this in praxis? The company should systematically develop these competencies in employees, using well prepared and focused training, communicating with them and giving them their own (top management) example. In addition, target competencies should be selection criteria when hiring and employing new staff. What is more, it is not enough to focus on employees. The company should also require such competencies from external partners.

One of the crucial areas is related to organizational culture improvements. It should support openness to change, the willingness to adopt new or innovative business models, and new production concepts. Therefore, companies should strive more toward flat organizations and enable organizational flow changes. They should strive toward the improvement of internal communication, enabling the knowledge and information to be shared among all key personnel as quickly as possible. Attention should be given to the company’s knowledge management, meaning a long-term and complex process of knowledge creation, transfer, and use. The next important aspect is related to motivation and the rewarding of individuals/employees, especially in the event of business success. When focusing on disruptive as well as other types of innovations, it is essential to accept certain risks and introduce a clear tolerance model for the failures. Special attention should be focused on improvements in the supply chain management. Obviously, the activities should be supported by appropriate budgeting. Last, but not least, trust among management and employees is one of the “hygienic” prerequisites for success.

The open innovation concept should also be implemented. Within this concept, special attention should be paid to cooperation with academia representing an important tool for achieving disruptive and breakthrough innovations.

As to marketing, companies should implement a dynamic market opportunities identification concept as well as provide shorter time-to-market. The research also stressed absolute focus on the customer as an important factor. It should be mentioned that such an approach can also be vague, as the company only focuses on fulfilling the customers’ needs. We think that such a concept can often kill disruptive innovations. Therefore, it is also important to develop breakthrough innovations which are not based directly on customers’ needs but have a clear market acceptance verification.

Enabling technologies should also be implemented, i.e., full digitisation at all company levels. One of these should be focused on the working process, especially within/after the Covid-19 experience. It is related to more virtual development and the adoption of working from home. In addition, artificial intelligence should be considered as a support to various business processes.

## Limitations of the Study

A possible limitation of the research is the homogeneity of the participants. It is related to the companies encompassed having different innovation and economic levels. In addition, there are differences between countries. Taking into account these differences, further studies would be welcome. In the future, it will be necessary to carry out studies in the field of SMEs in accordance with their innovation level, economic performance, and business sector. In addition, quantitative approaches would illustrate complementary aspects, but these require an appropriately higher number of respondents. Obviously, it will be necessary to focus on steps that enable the successful introduction of “real” disruptive innovations.

## Data Availability Statement

The raw data supporting the conclusions of this article will be made available by the authors, without undue reservation.

## Ethics Statement

Ethical approval was not provided for this study on human participants because the research was performed in accordance with relevant institutional and national guidelines. In Slovenia, the consent of ethical commission is required for other types of research (e.g., medical research). For social science research there is no such praxis. But the authors have to stress that our respondents were informed about the research goal and the fact, that their opinion will be used for the analysis and published anonymously. Only those respondents who have agreed with the aforementioned filled out the questionnaire. Written informed consent for participation was not required for this study in accordance with the national legislation and the institutional requirements.

## Author Contributions

VR: performance of all tasks, analysis, and basic text preparation. MM and BL: concept preparation, involvement in international data collection, writing of parts of text, and supervisory work. FP: concept preparation and supervisory work. All authors contributed to the article and approved the submitted version.

## Conflict of Interest

The authors declare that the research was conducted in the absence of any commercial or financial relationships that could be construed as a potential conflict of interest.
